# Epidemiology of Mastalgia in rural populations: A study from Ujjain, India

**DOI:** 10.6026/973206300212448

**Published:** 2025-08-31

**Authors:** Avneesh Arya, Banerjee G.K, Rubina Dohare, Sachin Parmar

**Affiliations:** 1 Department of Surgery, Government Medical College, Datia, Mathya Pradesh, India; 2 Department of Surgery, R.D.Gardi Medical College, Ujjain, Mathya Pradesh, India; 3 Department of Obstetrics and Gynaecology, Government Medical College, Datia, Mathya Pradesh, India; 4 Department of Community Medicine, V.K.S.Government Medical College, Neemuch, Madhya Pradesh India

**Keywords:** Mastalgia, fibroadenosis, fibroadenoma, breast pain, non-cyclical mastalgia, ultrasonography, FNAC

## Abstract

Mastalgia is a common breast complaint in women, particularly those of reproductive age, often leading to significant physical and psychological distress. Therefore, it is of interest to evaluate 100 female patients with mastalgia over an 18-month period, assessing them through clinical examination, ultrasonography (USG), fine needle aspiration cytology (FNAC) and mammography. The most frequent diagnoses were fibroadenosis (36%) and fibroadenoma (30%). Surgical management provided greater relief (90.5%) compared to medical therapy (80.9%), with Evening Primrose Oil being the most effective medical treatment. Thus, early diagnosis and appropriate therapy, including reassurance, are critical to effective management.

## Background:

Mastalgia, or breast pain, represents one of the most prevalent and distressing breast-related complaints among women of reproductive age, affecting their quality of life and causing significant psychological burden [1, 2]. This common condition manifests as breast pain that can range from mild discomfort to severe, disabling pain that interferes with daily activities, sleep patterns and sexual function [3, 4]. Despite its high prevalence and impact on women's lives, mastalgia remains underexplored, particularly in rural healthcare settings of developing countries like India, where access to specialized breast care services is often limited [5, 6]. The epidemiological significance of mastalgia varies considerably across different geographical regions and populations. In Western countries, mastalgia affects up to 70% of women during their lifetime, with prevalence rates ranging from 32% in the United Kingdom to 68% in the United States [7]. Asian populations, including India, demonstrate variable prevalence rates, with studies reporting between 16% in Sub-Saharan Africa to 51.5% in some Indian populations [8]. This geographical variation suggests important underlying differences in risk factors, healthcare access, diagnostic practices and cultural attitudes toward breast health that warrant further investigation. Mastalgia is traditionally classified into two primary categories based on its relationship to the menstrual cycle [9]. Cyclical mastalgia, accounting for approximately two-thirds of all cases, demonstrates a clear temporal relationship with hormonal fluctuations during the menstrual cycle, typically worsening in the luteal phase and improving with menstruation [10, 11]. This type predominantly affects women in their reproductive years and is attributed to normal physiological hormonal changes involving estrogen, progesterone and prolactin [1, 2]. Non-cyclical mastalgia, comprising the remaining one-third of cases, presents as continuous or intermittent pain unrelated to menstrual cycles and may indicate underlying pathological conditions including cysts, fibroadenoma, mastitis, or rarely, malignancy [9, 10]. The rural healthcare landscape in India presents unique challenges for understanding and managing mastalgia epidemiology. Rural women face multiple barriers to accessing specialized breast care services, including geographical distance from healthcare facilities, shortage of trained specialists, limited diagnostic equipment and socioeconomic constraints [12]. These factors contribute to delayed diagnosis, inadequate management and potential progression of underlying conditions that could be effectively treated with early intervention. Furthermore, cultural stigma, lack of awareness about breast health and reliance on traditional remedies often delay women from seeking appropriate medical care [13]. Recent studies examining rural-urban disparities in breast conditions have revealed important epidemiological differences that may influence mastalgia presentation and management [14, 15-16]. The diagnostic approach to mastalgia in rural settings requires adaptation to available resources while maintaining clinical effectiveness [17, 18-19]. Limited access to advanced imaging modalities such as mammography and MRI necessitates greater reliance on clinical examination and basic ultrasound evaluation. This resource constraint emphasizes the importance of developing locally appropriate diagnostic algorithms that can effectively differentiate between benign and potentially serious causes of mastalgia while minimizing unnecessary investigations and healthcare costs. Contemporary research emphasizes the need for population-specific epidemiological studies to understand mastalgia patterns in diverse healthcare settings [20]. By examining the epidemiology of mastalgia in rural Ujjain hospital, healthcare providers and policymakers can better understand the local disease burden, identify high-risk populations and develop culturally appropriate interventions to address this common but often overlooked women's health issue. Therefore, it is of interest to report the epidemiology of mastalgia in rural populations, specifically in the context of Ujjain.

## Materials and Methods:

## Study design and setting:

This was a prospective observational study conducted at the Department of Surgery, CRG Hospital, R.D.Gardi Medical College, Surasa, Ujjain (Madhya Pradesh), over a period of 18 months from January 2021 to June 2022. The hospital is located in a rural setting andSurasa is a village in Ghatiya tehsil of Ujjain district, characterized by a tropical climate.

## Study population and sample size:

A total of 100 female patients presenting with breast pain (mastalgia), with or without an associated lump, were enrolled in the study. These patients were treated either as outpatients or admitted as inpatients during the study period.

## Inclusion criteria:

Female patients presents with breast pain, regardless of whether the pain was cyclical or non-cyclical in nature.

## Exclusion criteria:

[1] Pregnant and lactating women.

[2] Patients with a previously diagnosed carcinoma of the breast.

## Consent process:

All participants were enrolled after obtaining informed written consent. Patients who declined to participate were not denied treatment. The purpose and nature of the study were explained to each participant in a language they could understand.

## Methodology:

All patients underwent a detailed clinical evaluation, including history and physical examination. Investigations included ultrasonography (USG), fine needle aspiration cytology (FNAC) and mammography where indicated. Baseline vital parameters such as pulse, blood pressure and ECG (for patients over 35 years of age) were recorded.

Initial management included medical treatment with:

[1] Paracetamol 500 mg

[2] Calcium supplements

[3] Capsule AD (Vitamin A and D)

[4] If unresponsive, patients were further treated with Capsule EVION 100 mg twice daily and Etizolam 0.25 mg

[5] In select cases, Evening Primrose Oil, Danazol, or Bromocriptine were administered.

An attempt was made to use a pain diary in a subset of educated patients; however, due to poor compliance, this approach was discontinued. Patients were randomly allocated to different medical management groups. Surgical intervention was performed when clinically indicated after preoperative evaluation. All procedures were carried out by qualified specialists.

## Monitoring and follow-up:

Patients were monitored regularly during treatment and follow-up visits. Clinical outcomes and treatment responses were documented in a structured proforma.

## Ethical considerations:

Ethical approval was obtained from the Institutional Ethics Committee (Certificate No. 40/2021). The study did not involve any invasive or hazardous diagnostic procedures, nor did it interfere with the standard treatment protocol. Confidentiality of participant data was strictly maintained throughout the study.

## Results:

The sample had 100 women patients who attended with mastalgia. Table 1 (see PDF) contains the demographic and lifestyle attributes of their respondents. Most patients (45%) belonged to the group 15-25 years, 35% of patients belonged to the group of 26-35 years. The majority of the respondents (76.3%) were rural based and 65.7 percent of them had non vegetarian food. On the issue of marital status, 62 per cent were married with 38 per cent being unmarried. Forty eight percent of the women gave a history of breast feeding and 52 percent were not breast fed, probably in relation to the number of unmarried women. It was revealed that unilateral pain occurred rather frequently (73%) than bilateral (27%) pain, as seen in Table 2 (see PDF). Dull aching pain (36%) was the most reported whilst continuous pain (21%) was the second-most reported pain. Other types of pain entailed intermittent (15%), throbbing (8%), pricking (4%) and non-specific dices (16%). In 42 percent of patients, a palpable breast lump accompanied with pain was seen and nipple discharge alone was observed in 7 percent of patients. As far as menstrual patterns are concerned, 67 percent had regular menstrual patterns. Non-cyclical (48%), cyclical (36%) and non-specific (16%) mastalgia were the liquids that defined the type of mastalgia. In age-wise distribution, 31R 40 age group showed the highest prevalence of cyclical or non-cyclical mastalgia (54%), whereas 26 percent of the women aged 41 to 50 years experienced the non-cyclical or non-specific mastalgia. Table 3 (see PDF) shows the clinical diagnoses, (ultrasonography) and FNAC results. Fibroadenosis (36%), fibroadenoma (30%) and non-specific breast pain (16%) were the most frequent clinical diagnoses. Relatively rare pathologies were breast abscess (8%), mastitis (5%), duct ectasia (4%) and breast carcinoma (1%). In 20 percent of the cases, fibrocystic changes on ultrasonography (USG) were observed and 29 percent showed fibroadenoma. Sixteen percent of the patients had normal breast tissue and 10% has not had an USG. Other findings were duct ectasia (4%), breast abscess (4%) and solitary mass (1%).FNAC was not done in 39 percent cases. Of the tested cases, 30 percent had fibroadenoma and 27 percent had fibroadenosis. Suspicious malignancy was also observed in 4 percent of cases. The results of management are provided in Table 4 (see PDF). Fifty-three patients were subjected to surgical intervention with 45 excisions and 8 incision and drainages (I \& D). Out of them 48 patients (90.6%) had relief, 5 patients continued to have pain. There were minor side effects (*e.g.* keloids formation) during 2 cases of excision. In 47 patients, medical management was given: 16 were administered all or some evening primrose oil (13 relieved). 7 were given Danazol (6 were relieved), NSAIDs were used to treat 14 (13 relieved). 10 were given placebo (counseled- 6 relieved). Danazol had side effects such as acne, muscle cramping and weight gain. Some NSAIDs brought about burning sensation, tingling and skin rash. There were no bad effects noted with both primrose oil and placebo. In general, the relief of mastalgia was reported in 86% patients. The efficacy between surgical management and medical treatment was only a bit different.

## Discussion:

The demographics and clinical profile of mastalgia in the rural Ujjain cohort show both concordance and divergence from earlier reports. In Ujjain, 45% of patients were aged 15-25 years, notably younger than the 30-50-year peak reported in a large case-control study of cyclic mastalgia (mean age ~36 years) [21]. The Ujjain study's predominance of non-cyclical mastalgia (48%)in a study from Bangladesh revealed that about one-third (35.5%) of the participants had experienced mastalgia [22] which is similar to our studywhile other in contrasts with the usual predominance of cyclical mastalgia, which accounts for 60-70% of cases in many series [23]. This shift may reflect differences in rural healthcare access, environmental exposures, or referral patterns. Unilateral pain was reported by 73% of Ujjain patients, with dull aching being most common (36%). By contrast, cyclic mastalgia typically presents as bilateral, diffuse, heavy pain radiating to the axilla and arms [21]. The Ujjain finding that 42% of patients presented with a palpable lump parallel a Turkish cohort, in which 48.8% reported pain plus lump, underscoring the frequent overlap of mastalgia with benign breast masses [24]. Ultrasound and FNAC in Ujjain confirmed fibroadenosis in 36% and fibroadenoma in 30%. These frequencies are similar to those in larger series: fibrocystic changes (42.2%) and fibroadenoma (21.1%) in 840 patients and fibroadenosis (46.3%) with fibroadenoma (20%) in another tertiary-center series [23, 24]. Such consistency reinforces the predominance of benign histologies in mastalgia. Management outcomes in Ujjain demonstrated 90.5% symptom relief with surgical intervention (excision and incision & drainage), with minimal complications and 80.9% relief with medical therapy. Among medical treatments, evening primrose oil (EPO) was best tolerated. In controlled trials, EPO's efficacy has been variable: one randomized trial showed no clear benefit over control oils (12.3% vs 13.8% reduction in pain days; P=0.73) [20], while observational data indicated clinically useful response rates of 41.2% with EPO versus 59.2% with Danazol (P<0.05) [25]. Topical NSAIDs achieved significant pain reduction in both cyclic and non-cyclic mastalgia, with nearly 50% of participants pain-free at six months and minimal side effects [26]. Danazol has consistently shown higher efficacy than non-hormonal options: a Peshawar cohort reported 77.8% relief with Danazol versus 55.6% with Panadol (P<0.004) [27]. Age-specific patterns in Ujjain more cyclical mastalgia in those <30 years and more non-cyclical pain in 31-50-year-olds are in line with hormonal pathophysiology of cyclic pain in younger women and the increasing likelihood of non-hormonal etiologies with age.

## Conclusion:

The non-cyclical mastalgia was more common among women of the reproductive age and was frequently complexed with benign breast disorders like fibroadenosis and fibroadenoma. FNAC and ultrasonography remains an essential key to diagnosis, particularly when palpable masses are present. Surgery managed better relief rates, but conservative therapies including Evening Primrose Oil, Danazol, NSAIDs, and Centchroman (where available) were also successful.
